# Comment on “Clinical Profile and Outcome of Japanese Encephalitis in Children Admitted with Acute Encephalitis Syndrome”

**DOI:** 10.1155/2014/767538

**Published:** 2014-08-27

**Authors:** Girish Chandra Bhatt, Tanya Sharma

**Affiliations:** ^1^Department of Pediatrics, All India Institute of Medical Sciences (AIIMS), Room No. 10, Saket Nagar, Bhopal, Madhya Pradesh 462024, India; ^2^Department of Pathology, All India Institute of Medical Sciences (AIIMS), Bhopal, Madhya Pradesh, India

We read with great interest the article by Kakoti et al. [1] and have the following comments to offer.

Out of 223 hospitalized acute encephalitis syndrome patients, 30% (67) were diagnosed as confirmed Japanese encephalitis (JE). In the recent studies enteroviruses (EVs) are being identified as one of the common causes of encephalitis in children worldwide [2, 3]. Various studies from India, Kuwait, and European countries report the prevalence of EV in encephalitis cases to be as high as 21-22% in encephalitis endemic area. Though the authors have tested samples for other flaviviruses such as dengue and West Nile viruses, it is surprising that these samples were not tested for the commoner enteroviruses.

Secondly, authors have nicely outlined the clinicodemographic profile of JE patients. However, it is surprising to find that the recently reported nonneurological manifestations of JE are lacking in the paper. Hepatomegaly, splenomegaly, deranged liver function tests, deranged renal function tests, thrombocytopenia, and so forth have been reported in JE patients [4, 5]. Some authors have suggested a possible change in virulence of JE virus or strain variation over time, developing properties similar to dengue like flaviviruses, responsible for these manifestations [5].
